# High-Throughput Single-Cell Manipulation in Brain Tissue

**DOI:** 10.1371/journal.pone.0035603

**Published:** 2012-04-20

**Authors:** Joseph D. Steinmeyer, Mehmet Fatih Yanik

**Affiliations:** 1 Department of Electrical Engineering and Computer Science, Massachusetts Institute of Technology, Cambridge, Massachusetts, United States of America; 2 Department of Biological Engineering, Massachusetts Institute of Technology, Cambridge, Massachusetts, United States of America; University of Antwerp, Belgium

## Abstract

The complexity of neurons and neuronal circuits in brain tissue requires the genetic manipulation, labeling, and tracking of single cells. However, current methods for manipulating cells in brain tissue are limited to either bulk techniques, lacking single-cell accuracy, or manual methods that provide single-cell accuracy but at significantly lower throughputs and repeatability. Here, we demonstrate high-throughput, efficient, reliable, and combinatorial delivery of multiple genetic vectors and reagents into targeted cells within the same tissue sample with single-cell accuracy. Our system automatically loads nanoliter-scale volumes of reagents into a micropipette from multiwell plates, targets and transfects single cells in brain tissues using a robust electroporation technique, and finally preps the micropipette by automated cleaning for repeating the transfection cycle. We demonstrate multi-colored labeling of adjacent cells, both in organotypic and acute slices, and transfection of plasmids encoding different protein isoforms into neurons within the same brain tissue for analysis of their effects on linear dendritic spine density. Our platform could also be used to rapidly deliver, both *ex vivo* and *in vivo*, a variety of genetic vectors, including optogenetic and cell-type specific agents, as well as fast-acting reagents such as labeling dyes, calcium sensors, and voltage sensors to manipulate and track neuronal circuit activity at single-cell resolution.

## Introduction

The brain is highly heterogeneous [1]–[3], and therefore requires single-cell resolution techniques for its analysis. Genetic manipulation, labeling, and tracking of single cells in brain tissues enable the analysis of neuronal circuits [4]–[6], cellular dynamics, and genetics in ways not possible using viral or other bulk methods [7]. Brain regions accessible via cranial windows *in vivo* and brain slice preparations *ex vivo* offer physically and optically accessible platforms in which to study single cells [4], [8]–[11], while at the same time maintaining the integrity of the tissue [12], [13]. Organotypic brain slices can be cultured for extended periods of time and serve as reliable platforms for studying the development and progression of disease and stress models [14]–[16], analyzing circuits in neural tissue [13], tracking axonal and dendritic morphologies [17]–[19], and developing pre-clinical models for various human diseases including Alzheimer's, stroke, and epilepsy [20]–[22]. Unfortunately, while optical microscopy techniques have been able to take advantage of the accessibility of brain slice cultures to achieve sub-cellular resolution imaging [23]–[25], technologies for genetically modifying and labeling cells with single-cell accuracy have been limited in both their speed and scalability. The researcher is left to choose between two groups of transfection techniques: one which enables cells to be transfected in bulk with little to no capability of targeting single cells, such as through viral labeling [26], [27], transgenics [28], [29], or biolistic transfection [30], or another group of techniques, that transfects cells with single-cell accuracy but at low-throughput and repeatability, such as manual microinjection [31], single-cell electroporation (SCE) [32]–[34], [19], or modified patch-clamping [5]. A high-throughput, scalable, easy-to-use, and reliable single-cell genetic manipulation technique could open new frontiers in neuroscience.

We developed a technology for high-throughput single-cell manipulation and transfection using computer-controlled servos, fluidics, and imaging which can rapidly move, clean, load, and target a front-loaded micropipette to single cells with micron-level accuracy and repeatability. The system requires no modification to currently established slice culture protocols [35], [36], [23] and is therefore compatible with and can greatly enhance previously established techniques for long-term sub-cellular imaging, electrophysiological recordings, and other experimental methods. Additionally, our system is not only cost effective but also compatible with standard liquid handling formats such as multiwell plates containing reagents to be transfected. After detailing the system design below, we demonstrate its operation through multi-colored labeling of neurons and also through combinatorial plasmid transfection of single cells in organotypic brain slices.

## Results

### System Design and Operation Overview

**Figure 1:**
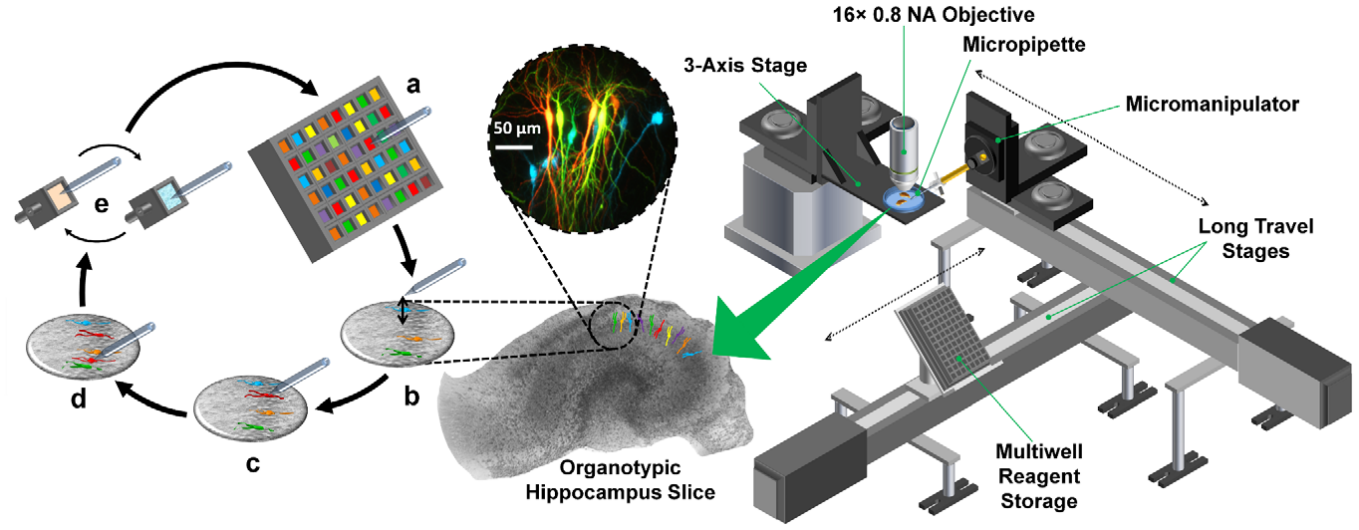
High-Throughput Single-Cell Electroporation System. The sequence of operations and the system are shown on the left and on the right, respectively. (a) The micropipette is front-loaded from a multiwell plate containing reagents, (b) before being rapidly transferred and positioned at a fixed location in the field of view of the microscope objective. (c,d) The three-axis stage holding the slice bath/manipulation chamber moves the sample and bring cells into contact with the stationary micropipette, allowing transfections to be carried out. (e) The system automatically transfers the micropipette tip to a cleaning solution bath as well as a rinsing reservoir where the tip is washed and prepped to sample a different well of the multiwell plate, allowing the cycle to continue. Pyramidal cells depicted in inset were imaged for Cerulean, EGFP, YFP, and tdTomato 24 hours post-transfection.

The key features of our system are shown in Figure 1. (a) A micropipette acts as a short-term diffusion-restricted sample reservoir after drawing in small volumes of reagents from a multiwell plate. (b) The micropipette is automatically transferred to the tissue slice bath and brought into a fixed point in the user's field of view. (c,d) By using a stage controller in conjunction with fluorescence imaging of dye outflow from the micropipette and phase-contrast imaging of cell soma, the user can rapidly bring cells into contact with the micropipette and electroporate them using an electrical pulse sequence we designed for reliable high-efficiency transfection. (e) When transfection of a reagent is completed, the system automatically removes, cleans, and washes the micropipette before beginning the transfection cycle of a new reagent. In this way, the same micropipette can be used to transfect many cells with multiple reagents within the same brain slice.

### Single-Cell Electroporation using Front-Loaded Micropipettes

Single-cell electroporation (SCE) has emerged as a versatile means for transfecting cells due to its potential for high efficiency [37], [33], its ability to transfect a variety of agents including dyes [38], plasmids [8], and RNAi reagents [39], and its tissue and *in vivo* compatibility [9], [8], [40]. The fundamental operation of the technique relies upon loading a micropipette with a transfection reagent, which may contain a mixture of multiple agents such as plasmids, in an ionic solution, and then positioning the tip opening on or near the cell of interest before applying an electrical signal to electroporate the membrane of the targeted cell. The micropipette therefore serves as both a highly-focused electrode and a sample delivery device. In all publications to date, however, micropipettes have been used in a disposable, short-term manner in which they are pre-loaded through manual backfilling with the reagent mixture immediately before usage. Each micropipette is used to transfect only one loaded sample or mixture, and transfection of a different sample or mixture necessitates exchanging the micropipette. This is time-consuming and therefore has limited high-throughput applications of SCE. In addition, use of multiple micropipettes, even when pulled under similar conditions, introduces significant variability in transfections. No clear strategy has been presented to date as a means of expanding conventional SCE so that it could be scaled for high-throughput purposes.

**Figure 2:**
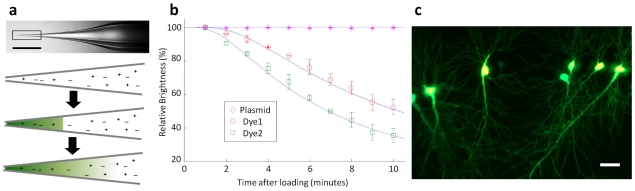
Single-Cell Electroporation Can Be Carried Out at High Efficiency by Front-Loading Micropipettes. (a) In a micropipette filled with saline (scale bar 1 mm), a front-loaded reagent diffuses over time, decreasing concentration of the sample at the tip (drawings are not to scale). (b) Approximately 2 nL of three different fluorescent molecules, Alexa Fluor 594 (Dye1) and 488 (Dye2) hydrazide salts and SYBR-Green-labeled pEGFP-N1 (Plasmid), were front-loaded into micropipettes and their fluorescence monitored at 1 minute intervals over ten minutes and compared to simulations (continuous lines). Each data point is the mean ± s.d. of three independent experiments. (c) Concentration of plasmid in the tip of the micropipette remains stable enough to reliably transfect multiple cells with pEGFP-N1. 79.1±8.7% of the transfected cells expressed EGFP 24 hours following transfection (*n* = 72 from six independent experiments, where 12 cells were transfected within 2 minutes in each experiment). Scale bar 50 µm.

To address this, we investigated whether it is possible to front-load small volumes of reagents (on the order of nL) into a micropipette containing a standard electrically conductive solution while maintaining accurate knowledge of reagent concentration at the tip. While for microinjection, a front-loaded reagent can be isolated at the tip via an air-gap in the micropipette, for electroporation, a continuous conductive salt solution must exist to maintain electrical continuity from the electrode (generally Ag/AgCl) to the volume of the reagent at the tip. Consequently, any reagent front-loaded into a micropipette will diffuse and dilute into the greater volume of the salt solution over time (Fig. 2a). We found however that the micron-scale tip dimensions of micropipettes in combination with the large molecular weights (and correspondingly low diffusion coefficients) of plasmids, can provide a means of maintaining a relatively non-diffuse and stable volume of reagent at the tip over time scales of several minutes where the high speed of our semi-automated system allows many cycles of single-cell transfections. Micropipettes loaded with 5 µL of Ringers solution were front-loaded with approximately 2 nL (−30 psi applied for 15 seconds) of either Alexa Fluor 594 hydrazide, Alexa Fluor 488 hydrazide, or SYBR-Green-labeled 4.7 kbp plasmid (pEGFP-N1) and were then immediately inserted into a saline bath where we monitored the brightness of the fluorescence in the tip over time to infer concentration changes. Using empirically determined molecular parameters from the literature (see Methods), we also carried out simulations of the diffusion process for the different species and compared these to our experimental data (Fig. 2b, Fig. S1). While low molecular weight species (the Alexa Fluors) diffused away quickly, the concentration of plasmid at the tip varied by only a few percent over the ten minute duration of the experiments. Therefore, using a high-speed platform, front-loaded micropipettes can maintain stable concentrations of plasmids for sufficient amounts of time to enable sequential electroporation of single cells.

We experienced inconsistent and inefficient transformation efficiencies using standard SCE electrical pulse parameters reported in the literature [33], [9] and therefore screened a variety SCE pulse parameters, including different pulse repetition frequencies and pulse duty cycles using our platform. Examples of transfection efficiencies with different pulse parameters are shown in Table S1. We found that a short 100 millisecond burst of −10 V 1 kHz (10% duty cycle) provided the highest efficiency in our system compared to more commonly used lower repetition rate pulses with long total durations such as a 200 Hz (20% duty cycle) or 50 Hz (2.5% duty cycle) repetition rate for a one second duration. Using these pulse parameters with front-loaded micropipettes, we transfected cells in the pyramidal cell layer (PCL) of the CA1 within approximately 40 µm of the surface of organotypic hippocampal slices with 300 ng·µL^−1^ pEGFP-N1 and 50 µM Alexa Fluor 594 hydrazide for visualization in standard Ringers solution. At 24 hours following electroporation (Fig. 2c), the transformation efficiency as determined by EGFP expression was 79.1±8.7% with very high repeatability (*n* = 72 cells from six independent experiments), comparable to the best efficiencies reported in conventional SCE methods [33], [9]. It should be noted that in these experiments, no expression of fluorescent protein was observed in any of the cells that were not targeted for electroporation. Additionally, expression of plasmids in cells was consistently long-term, with sufficient expression in cells at 7 days post-transfection for both dendritic spine counting as well as neurite morphology analysis (Fig. S2).

### Micropipette Tip Recycling

We next investigated if micropipettes can be completely cleaned of reagents, and subsequently reloaded without any cross-contamination, therefore allowing the transfection of multiple reagents. During standard use, micropipettes frequently clog with debris, an issue which can be monitored by visual analysis of dye outflow at the tip and by measurement of the electrical conductivity of the tip. The major contributing factors to clogging are debris arising from the cell-to-micropipette contact and the precipitation of plasmids at the tip, both of which greatly inhibit the ability of a micropipette to be flushed beyond several sample-rinse cycles. We therefore developed a robust method for cleaning the tip by immersing the tip of the micropipette into a well containing a continually-perfused (1.5 mL·min^−1^) solution of 0.25% sodium hypochlorite solution and by applying 20 seconds of alternating +30 psi and −30 psi gauge pressure pulses with a 2∶1 positive/negative duration ratio. The resulting net positive flow out of the tip ensures that sodium hypochlorite is not left in the conductive saline bridge of the micropipette. Immediately following the cleaning step, the micropipette is withdrawn and the tip is then inserted into a well containing a continuous perfusion of deionized water (1.5 mL·min^−1^). +30 psi is then applied to the micropipette for five seconds to further ensure remaining sodium hypochlorite is removed. Using this technique, the micropipette could be successfully cleaned and reloaded 92±3.2% of the time (*n* = 100, based on 25 sequential load-clean-rinse cycles from four independent experiments).

**Figure 3:**
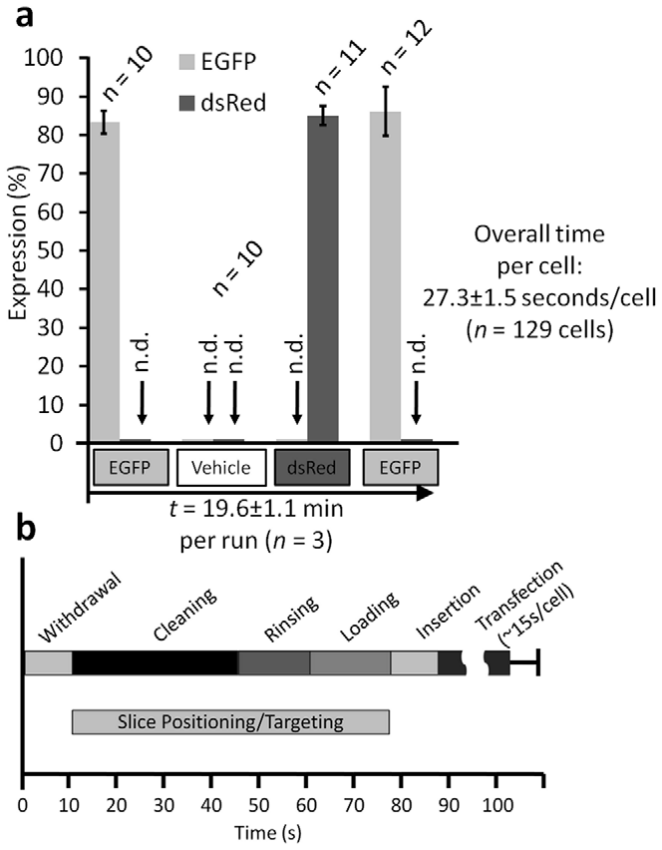
Rapid Transformation of Single Cells with Different Reagents with No Cross-Contamination. (a) Sequential transfections were carried out on groups of single cells using the same micropipette, in which pCAG-EGFP was transfected first (efficiency: 83.3±2.9%), followed by a reagent containing only the fluorescent vehicle solution (plasmid contamination: 0%), then pCAG-dsRed (efficiency: 85±2.9%, plasmid cross-contamination: 0%), and finally pCAG-EGFP again (efficiency: 86.1±6.4%, plasmid cross-contamination: 0%). The entire sequence took 19.6±1.1 minutes. (n.d. = not detected) Results are mean ± s.d. of three independent experiments in which 129 total cells were transfected. (b) Average time of different steps in a full transfection cycle. Withdrawal, cleaning, rinsing, loading, and insertion to slice bath of the micropipette takes 88.9±8.6 seconds (from four independent experiments, n = 25 per experiment). Transfection/dye uptake of single cells takes on average 14.8±6.2 seconds per cell (n = 56 from five separate experiments).

To characterize how capable our cleaning methodology is at minimizing cross-sampling (from residues of previous loadings), we next performed a sequential four-part transfection experiment on cells of the CA3 PCL which consisted of pCAG-EGFP, then vehicle, then pCAG-dsRed, and then pCAG-EGFP. At 36 hours post-transfection, cells were analyzed for fluorescent expression. We observed 86.1±6.4% overall efficiency (n = 99 cells) with 100% sample specificity (0% cross contamination). Even when all system operations (including loading, targeting, electroporating, washing) are taken into account, it takes only 27.3±1.5 seconds per cell (n = 129 cells) (Fig. 3a).

### Automation, Control, and Throughput

We developed a comprehensive software suite in MATLAB and the C programming languages for precision control and synchronization of all aspects of the system automation through a National Instruments Data Acquisition (NIDAQ) card and USB/Serial communication (Figs. S3, S4, and S5). While the user selects the cells to be targeted and transfect, a single high-level control allows triggering of the system to withdraw, clean, rinse, reload, and finally reposition the micropipette. Long-travel servos move the entire micromanipulator at 100 mm/s between the slice bath and the tip cleaning/reagents, which minimize chances for clogging due to exposure to air. A computer-controlled bank of valves as well as pressure and vacuum regulators then precisely rinse, clean, and load the micropipette. Each full transfection cycle (including cleaning and loading) takes only 88.9±8.6 seconds (Fig. 3b), (*n* = 100 from four independent experiments of 25 transfection cycles each). The targeting and transfecting of individual CA1 and CA3 pyramidal cells generally requires on average 14.8±6.2 seconds per cell (n = 56).

### Long-Term Operation

We found that commonly used Ag/AgCl wire electrodes are not compatible for long durations of operation because the repeated hyperpolarizing pulses eventually release Ag^+^ ions into solution even after the wire had been properly chloridized, resulting in variation in electrical conductivity. The presence of Ag^+^ ions in solution is also potentially toxic to cells. Critically, the Ag^+^ ions in solution also react with sodium hypochlorite during the washing step to form AgCl in the proximity of the micropipette tip, which precipitates leading to the clogging of the tip. To avoid these shortcomings, we used an electrode holder containing a 30 American Wire Gauge (AWG) platinum wire that does not degrade. We found that electrical resistance (measured with a hyperpolarizing 5V DC voltage) to vary by only about 3% per hour of usage (*n* = 10 micropipettes from 10 independent experiments).

With all of these developments, we were able to continuously recycle and use a single micropipette for over six hours. Only the amount of solution initially backfilled into the micropipette limited this operation duration because each transfection cycle results in a net loss of saline in the micropipette. A micropipette loaded with 6 µL of solution was capable of providing three hours or more of operation, and when initially filled with 12 µL, six hours of continuous usage was possible.

### Multicolor Combinatorial Labeling of Single Cells within Brain Slices

**Figure 4:**
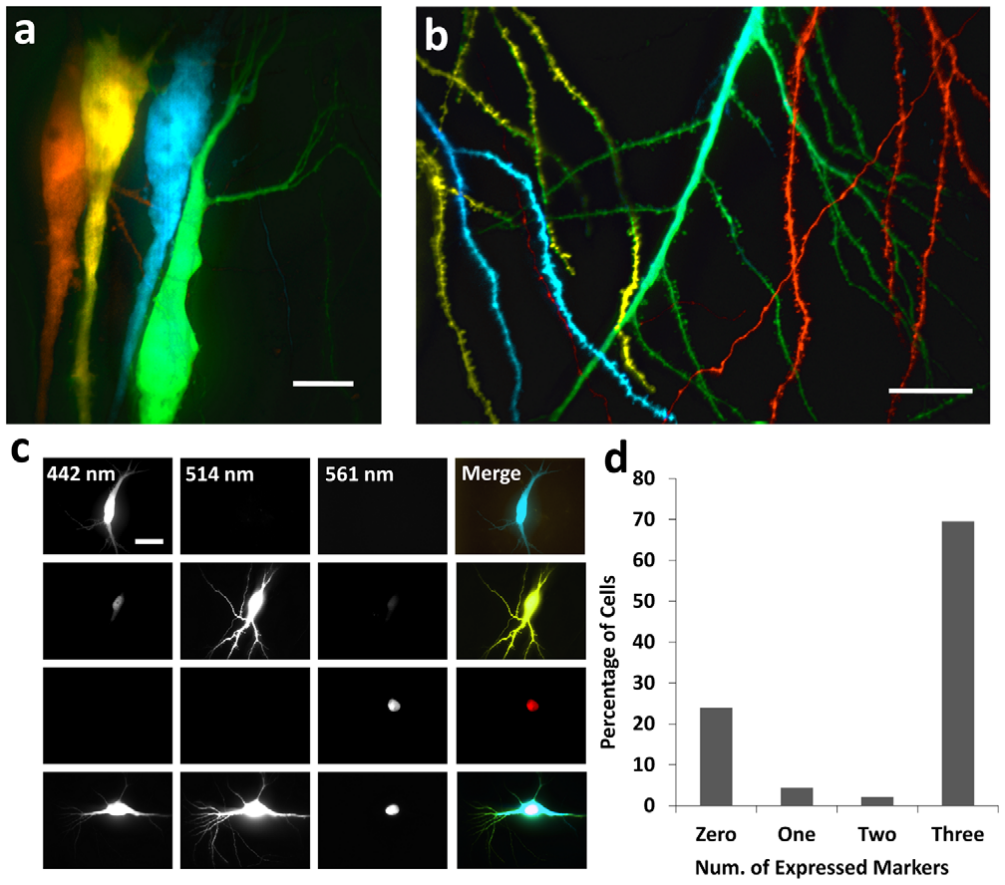
Multicolor Labeling of Single Cells within Brain Tissues and High Co-transfection Efficiencies. (a) Multiple fluorophore-encoding plasmids (pCI-tdTomato, pCAG-YFP, pCAG-Cerulean, and pCAG-EGFP) are transfected into neighboring cells in the same slice. (b) Overlapping processes can be easily distinguished. (c) pCAG-Cerulean, pCAG-YFP, and mCherry-Lac-Rep express three spectrally distinguishable fluorescent markers: cytosolic 442/470 nm (ex/em), cytosolic 514/530 nm (ex/em), and nuclearly-localized 561/610 (ex/em), respectively, as shown in the top three rows. They were used to determine co-transfection efficiencies of multiple plasmids, as shown in the bottom row. (d) A mixture containing 300 ng·µL^−1^ of both pCAG plasmids and 347 ng·µL^−1^ of mCherry-Lac-Rep was transfected using our front-loaded SCE methodology into CA1 and CA3 pyramidal cells in organotypic hippocampal slices and imaged 24 hours later for expression patterns. 92 cells total were transfected, with 23.9% expressing no visible fluorescence signal, 4.3% expressing only one type of fluorescent protein, 2.2% expressing only two types of fluorescent proteins, and 69.5% expressing all three types of fluorescent proteins. Scale bar in panel a is 10 µm, and in b and c is 20 µm.

The ability to uniquely label individual cells permits tracking and analysis of multiple cells within complex brain tissues. The “brainbow” technique [29] is a pioneering method to achieve multicolor single-cell labeling, however the need to engineer transgenic animals, and the density and the stochasticity of labeling limits its wide-range use. The capability to rapidly and deterministically label single cells with multiple combinations of colors and without using transgenic animals provides significant flexibility. Using our system, we were able to transfect multiple adjacent pyramidal neurons within minutes of one another (Fig. 4a) with different fluorescent reporter plasmids (Methods Section). We were also able to easily differentiate the dendritic processes of multiple neurons (Fig. 4b).

Testing effects of multiple genetic perturbations in tissues while simultaneously labeling cells with fluorescent reporters for tracking, traditionally requires the development and use of specialized vectors expressing multiple proteins. However, because SCE can transfect mixtures of multiple plasmids, we proposed that mixing plasmids encoding genes of interest along with known fluorophore-encoding plasmids would allow for a means of rapidly analyzing effects of these genes. In addition, this same methodology could allow co-transfection with multiple fluorescent reporters enabling the labeling and differentiation of greater numbers of densely packed cells in a given brain slice. To ensure co-transfection could reliably be performed using our modified SCE techniques, we transfected cells with a sample comprised of 1∶1∶1 molar ratios of three plasmids: pCAG-Cerulean, pCAG-EYFP, and mCherry-Lac-Rep (nuclearly-localized mCherry), at concentrations of 300 ng·uL^−1^, 300 ng·uL^−1^, and 347 ng·uL^−1^, respectively, as well as 50 µM Alexa Fluor 594 in Ringer's solution (Fig. 4c). 92 CA3 pyramidal cells were electroporated in eight different organotypic slices with this three-plasmid mixture. 24 hours following transfections, fluorescence was observed in 70 of the electroporated cells. Of these 70 cells, 64 (91%) expressed all three fluorescent signals, while 2 cells (2.8%) expressed only two at significant levels, and 4 cells (5.6%) expressed only one fluorescent maker, indicating a very high triple-transfection rate for multiple plasmids (Fig. 4d).

### Combinatorial Genetic Modification of Single Cells within Brain Slices

The ability to rapidly genetically modify single cells can allow parallel analysis of many genetic modifications in a single brain slice. The protein Kalirin is a Rho guanine nucleotide exchange factor (RhoGEF) which exists as a number of alternatively spliced isoforms in the mammalian brain [41], and its functions have been investigated in a series of elegant papers [42]–[49]. The exogenous expression of several Kalirin variants, notably Kalirin-7, has been shown to significantly modify dendritic spine morphology in cultured cortical neurons [50] as well as hippocampal interneurons [46]. We selected three plasmids encoding Kalirin-5, Kalirin-7, and Kalirin-9 with a myc-tag (see Methods) for use in a combinatorial fluorophore plasmid test on linear dendritic spine density in cells of the CA3 PCL.

To check whether the labeling with different fluorophores introduce bias in the observed linear spine density, we transfected a total of 120 cells in the CA1 and CA3 PCLs with the four different fluorophore-encoding plasmids to be used. Linear spine densities of the basal dendritic arbors of the cells were counted for each population of cells at 24 hours post-transfection (*n* = 120 cells total) (Fig. S6). No statistical difference in spine density among the populations of cells transfected with the fluorophores Cerulean, EGFP, YFP, and tdTomato was observed. (*F_crit_* = 2.63, *F* = 0.29 and 0.62 for CA1 and CA3, respectively).

**Figure 5:**
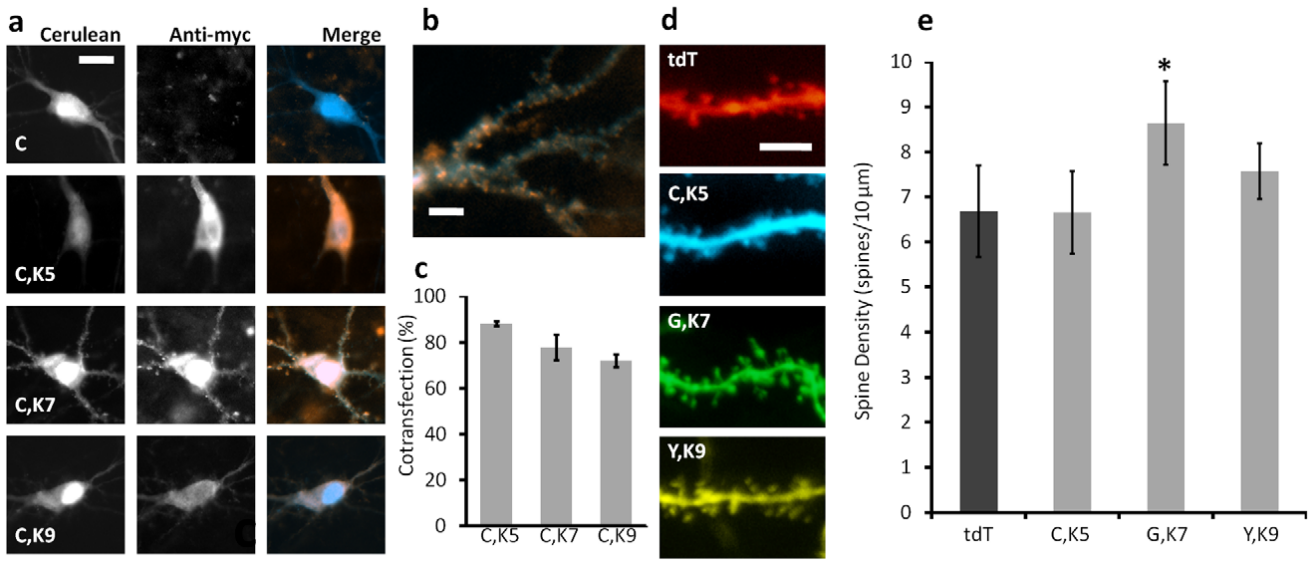
Combinatorial Genetic Transformation of Multiple Single Cells within Brain Tissue and Effects on Spine Densities. (a) Plasmids encoding the three isoforms of Kalirin (Kalirin-5, Kalirin-7, and Kalirin-9) were each co-transfected with pCAG-Cerulean by our system into CA3 pyramidal cells in hippocampal slices. 24 hours following transfection, slices were fixed and stained for the myc tag from the Kalirin isoforms before imaging for Cerulean (cyan) and myc (orange). Scale bar 15 µm. (b)Anti-myc staining in Kalirin-7-transfected cells shows localization into fine points on the dendrites. Scale bar 5 µm. (c) Co-transformation efficiencies determined by co-staining for pCAG-Cerulean and the three pEAK-His-Myc-Kalirin vectors, were 88.2±2.2% (*n* = 24), 77.1±11.1% (*n* = 21), and 71.9±5.5% (*n* = 20) for Kalirin-5, Kalirin-7, and Kalirin-9, respectively. (d) A series of transfection cycles were carried out on CA3 pyramidal cells in the same slice (8 total slices) to analyze the effects of exogenous expression of the three Kalirin isoforms on linear dendritic spine density of basal dendrites. Cells were transfected with either tdTomato (tdT) as a control, Cerulean and Kalirin-5 (C,K5), EGFP and Kalirin-7 (G, K7), or YFP and Kalirin-9 (Y, K9). Spines were imaged 24 hours post-transfection. Scale bar is 10 µm. (e) Linear spine densities for four groups: tdT cells had an average of 6.7±1.0 spines/10 µm (*n* = 95 segments from 19 cells). C,K5 exhibited no statistically higher spine density (6.65±2.0 spines/10 µm, *n* = 60 segments from 11 cells, p = 0.74) and so Y,K9 (7.6±0.6 spines/10 µm *n* = 55 segments from 9 cells, p = 0.08). G,K7, however, did show a statistically higher linear spine density (*) compared to tdT (8.6±0.9 spines/10 µm, *n* = 75 segments from 15 cells, p<10^−4^).

To verify successful expression of the Kalirin isoforms, each plasmid was co-transfected along with pCAG-Cerulean into CA3 pyramidal cells in organotypic slices. 24 hours following transfections, slices were fixed, stained, and imaged for myc-tag and Cerulean expression (Fig. 5a). Differences in cell staining patterns were readily apparent, with the cells transfected with Kalirin-5 and Kalirin-9 exhibiting myc staining in the cytosol, while cells transfected with Kalirin-7 exhibiting localized myc-labeling in the dendrites (Fig. 5b), in agreement with the evidence for Kalirin-7 localization to the post-synaptic densities due to its Sec14p/spectrin-like repeat region unique amongst the Kalirin isoforms [47]. Co-transfection rates, determined by co-localized Cerulean fluorescence and anti-myc staining, were 88.2±2.2% (*n* = 24), 77.1±11.1% (*n* = 21), and 71.9±5.5% (*n* = 20) for Kalirin-5, Kalirin-7, and Kalirin-9, respectively. (Fig. 5c).

We next transfected individual cells in the CA3 PCL with one of four different plasmid mixtures: tdTomato (control), Cerulean/Kalirin-5, EGFP/Kalirin-7, and YFP/Kalirin-9. Linear spine densities of the basal dendrites were analyzed 24 hours post transfection (Fig. 5d). Because each isoform was co-transfected with a known and unique fluorophore, we could readily distinguish and analyze cells that had been transfected with different Kalirin-isoform-encoding plasmids in the same tissue slice even for neighboring cells with significantly overlapping processes. When compared to the control cells with an average of 6.7±1.0 spines/10 µm (*n* = 95 segments from 19 cells), no statistical difference in linear spine density was observed in Kalirin-5-transfected cells (6.65±2.0 spines/10 µm, *n* = 60 segments from 12 cells, p = 0.74), as well as Kalirin-9-transfected cells (7.6±0.6 spines/10 µm *n* = 55 segments from 11 cells, p = 0.08). However, a statistically significant difference in linear spine density was measured in Kalirin-7-transfected cells (8.6±0.9 spines/10 µm, *n* = 70 segments from 15 cells, p<10^−4^) (Fig. 5e). Our results are in agreement with the previous studies on the effects of Kalirin isoforms [42]–[50], which suggest that our methodology based on co-transfection of multiple plasmids provide statistically significant results in a high-throughput, single-cell manner.

Acute hippocampal slices were also tested, in order to assess the system's operational characteristics in a tissue environment more closely resembling *in vivo* conditions (Fig. S7). Because plasmid expression in acute slices cannot be compared directly to expression in organotypic slices due to the limited lifetime of acute slices (several hours), we compared the cell targeting and electroporation efficiency of our system between acute and organotypic slice cultures, which we define as the percentage of cells intentionally and successfully electroporated and filled with fluorescent dyes (determined visually by dye uptake). No significant difference was found between the two tissue cultures: In acute slices, the targeting efficiency was 95.3±4.2% (*n* = 62 in five separate experiments) while in organotypic slices it was 96.0±5.4% (*n* = 56 in five separate experiments). No off-target electroporation, which we define as the electroporation of unintended adjacent cells, was observed in either case. The higher density of cells in acute slices made the average cell-targeting time longer however: In acute slices, it took 26.5±8.9 seconds per cell, (*n* = 62 cells in five separate experiments) while it took 14.8±6.2 seconds per cell in organotypic slices (*n* = 56 cells from five separate experiments) to achieve the level of electroporation efficiency reported above. This significant difference in timing (p<10^−5^) was primarily due to the extra time needed in targeting cells with less-clearly defined somatic boundaries in acute slices, as well as the slower movement of the micropipette in acute slices. These results were expected, based on the often-observed flattening and thinning of organotypic slices when compared to acute slice environments [13].

## Discussion

The diversity of cells and the complexity of neuronal circuits in the nervous system require single-cell resolution studies. However, single cell studies have so far been painstakingly slow and error-prone. Here, we demonstrated a technology which permits single cells to be genetically manipulated rapidly inside brain tissue, enabling significant acceleration of the throughput of standard single-cell analytics and techniques used on brain slices. We also designed this system to be low cost and compatible with standard brain slice culture equipment and techniques to make it readily adaptable by the research community.

Our system can be readily applied to both organotypic and acute slice formats (Fig. S7), the two primary tissue culture methodologies. A wealth of research exists in using brain slice platforms, and particularly organotypic cultures, in modeling many human diseases including Alzheimer's, Parkinson's, and epilepsy, all of which could benefit from the increased throughput in single-cell manipulation and analysis [21], [22]. In addition to plasmids encoding cDNA, shRNA-encoding plasmids can also be transfected as well as both long coding RNA and siRNA using SCE [51], [39]. Optogenetic proteins could also be transfected with single-cell resolution [52], [53]. The throughput of our technology also makes possible the use of brain slices in high-content, single-cell resolution screens. For instance, large libraries of cDNA or RNAi encoding vectors could be rapidly tested for their effects on neurite and synaptic morphogenesis in brain tissue. Fast-acting reagents such as multiplexed fluorophores, calcium-sensing, and voltage-sensing dyes can also easily be transfected via SCE into both acute and organotypic slices using our system providing a means of real time connectivity and circuit analysis at cellular resolution [54]. Additionally, we can also sequentially transfect reagents into the same cells, enabling pre-and-post transfection analysis [18] (Fig. S8). Furthermore, it is also feasible that our system can be used in conjunction with single-cell electrophysiology techniques using conventional micropipettes and labeling as shown by Rancz et al. or with novel nanoprobe electrical recording techniques demonstrated by Qing et al. [55], [5].

Cell-type-specific transfections could be carried out by using either (a) tissues from transgenic animals that express fluorescent reporters driven by cell-specific promoters, or (b) wild-type tissues labeled with fluorescent reporters (driven by cell-specific promoters) delivered using bulk transfection through viral or biolistic techniques. Once the subpopulations of cells are labeled with fluorescent reporters, their identification, targeting, and transfection with reagents is readily possible with our platform. Additionally, since larger plasmids (up to 13 kbp were transfected in this paper) can be introduced using SCE, cell-type specific promoters can be incorporated into transfected vectors in order to add a further level of specificity to our system.

Our system could in principle be adapted for deeper tissue and even *in vivo* single-cell manipulations using cranially accessible preparations and multi-photon microscopy [8]–[11]. When working at greater depths *in vivo*, the speed of the system would need to be decreased in order to avoid damage to both tissue and micropipette as we did for acute slices above. However, because *in vivo* preparations can be operated on over longer time periods than acute brain slices, it should still be feasible to perform large-scale *in vivo* single-cell manipulations through cranial window preparations. By enabling more variables to be tested within the same tissue and on specific anatomical regions, the effect of variability between multiple tissue preparations and between animals can be avoided. More efficient utilization of tissues could also enable larger scale studies.

## Materials and Methods

### Single-Cell Electroporation

Micropipettes were pulled from 1.2 mm OD, 0.60 mm ID filament capillary glass (Sutter) to an opening diameter of approximately 1.0 µm on a Sutter P-97 Flaming-Brown puller with a 2.5 mm×2.5 mm box filament (FWB255) at settings RAMP = 490; HEAT = 490 PULL = 0 VEL = 24 TIME = 250 (4 Loops). Ringers solution (135 mM NaCl; 5.4 mM KCl; 0.5 mM MgCl_2_; 1.8 mM CaCl_2_; 25 mM HEPES) was balanced to pH 7.4, and 6 µL of the solution was back-filled into the micropipette using a gel-loading pipette tip (Invitrogen). The micropipette was mounted on a holder (WPI) modified to use 30 AWG platinum wire (Alfa Aesar) for its electrode. Micropipette resistances at DC were approximately 8 to 10 MΩ for experiments in this paper. For electroporation, the electrode was driven directly from a pCIe-6259 National Instruments Data Acquisition Card (NIDAQ), which enabled both hyperpolarizing and depolarizing pulses with magnitudes up to 10 V. For signals with a larger magnitude, an amplifier with ±18 V power supply rails driven by the NIDAQ card was used. During targeting of cells for transfection by the user, bright-field/phase-contrast illumination was used to coarsely move the desired cell soma near the micropipette tip. Following this, epi-fluorescence visualization of Alexa Fluor 594 or 488 dye was used to bring the tip into fine contact with targeted cells. By monitoring the outflow of the fluorescent dye, and noting when it almost ceased, we were able to reliably find a location at which to electroporate cells. Low pressure (+1 psi) was applied to the micropipette to prevent clogging as it approached the cells. Just before applying the electrical pulse train, the system automatically released pressure. Pressure was not reapplied until tip was removed from proximity of cell.

### System Automation, Control Software, Electroporation Equipment, Pressure Control

All Sutter instruments were interfaced using custom-written code in C/C++, while the long-travel stages (ROBO Cylinders by International Automation Incorporated) were controlled through the serial port interface using standard protocols. The Data Acquisition Toolbox in MATLAB was used for controlling the PCIe-6259 NIDAQ card. A computer-controlled bank of electrical valves (Numatics) selectively apply one of five preset positive and negative gauge pressures to the gasket/holder assembly for purposes of cleaning, rinsing, loading, and transfection. The software and most up-to-date drivers and operating system requirements are available upon request from the authors (M.F.Y.).

### Imaging and Analysis

A 16×0.8 NA water-dipping objective was used on an FN-1 upright electrophysiology microscope (Nikon), utilizing bright field and epi-fluorescence. A Hitachi KP-M2RU near-infrared monochrome CCD camera was used in conjunction with either a Nikon TRITC HQ cube or a FITC HQ (both Nikon). A multi-focal Visitech vtHawk confocal imaging unit, CoolSnap HQ camera, and PIFOC-400 400 µm travel piezo were used for high-speed imaging of cells after transfection. For spine imaging, a 60×1.0 NA objective (Nikon) was used, while for lower resolution/magnification images, the 16×0.8 NA objective was used. When collecting data for dendritic spine analysis, z-stack slices were taken at 0.5 µm increments, and for low-magnification images z-stack slices were taken at 2 µm increments. For immunohistochemistry imaging, we used a TE-2000 microscope in conjunction with either a 20×0.7 NA or 60×1.4 NA oil immersion objective and Nikon Elements Advanced Research. Because all images were monochromatic, prior to analyzing spine densities, image files were fed into a custom-written MATLAB script which both renamed randomly and recorded the original name of each image file to ensure blind analysis. Following image analysis, the files were matched up with their encoded names in order to properly compare data. Spine counting was conducted manually in a blind fashion.

### Organotypic Slice Culture and Acute Slice Harvesting

P5 to P9 Sprague-Dawley rat pups were sacrificed and their hippocampi were sliced immediately at 300 to 350 µm thickness using a Vibratome and cultured on membranes (Millipore PICM0RG50) as described previously [36]. Slices were kept for up to six weeks in the case of organotypic culture, or immediately transferred to the working slice bath, in the case of acute slices. All animal work was approved by the MIT Committee of Animal Care and Division of Comparative Medicine and abided by institutional, state, and federal guidelines for animal welfare. For organotypic work, slice media was changed 24 hours following slicing and every third day afterwards. To avoid contamination, organotypic slices were rinsed in pre-warmed Rat Ringers Solution (buffered to pH 7.4) containing 100 U/mL penicillin and 100 µg/mL streptomycin and were returned to a well containing fresh media, containing both containing antibiotics at above concentration and 60 ng/mL of Nystatin immediately following either transfections or imaging in the slice bath. Using this methodology as well as standard techniques during slicing, contamination of organotypic cultures was extremely rare. For transfections in acute slices, the bath chamber was perfused with warmed Rat Ringer's solution that was continuously bubbled in carbogen (95% O_2_/5% CO_2_). Acute slices were maintained for up to three hours.

### Immunohistochemistry

For staining slices, the entire membrane inserts were rinsed in Tris-Buffered Saline and Tween-20 (TBST) for five minutes, followed by fixing for 10 minutes at room temperature in 4% Paraformaldehyde in Phosphate Buffered Solution (PBS). TBST was introduced to the fixing solution before aspirating and rinsing twice in TBST. Slices were permeabilized in 0.1% Triton X-100 for 10 minutes at room temperature before rinsing twice in TBST, and then incubated in 1% casein in TBST for 60 minutes at room temperature. Slices were then cut out of their membrane inserts and placed into 24 well plates and incubated with antibody (anti-myc conjugated to Alexa Fluor 555 from Millipore) in TBST containing 0.4% casein for three hours at room temperature. Slices were then rinsed in TBST with rocking for thirty minutes changing TBST every ten minutes before being mounted on slides in Vectashield under cover glass, sealed with nail varnish, and being stored at 4 degrees Celsius in the dark. Using the described protocol we did not see significant loss of native fluorescence in Cerulean and therefore did not need to use anti-bodies for its imaging.

### Plasmids and Sample Preparation

Plasmids were grown in the conventional bacteria strains XL-1 Blue, DH5α, or TOP10. All plasmids were harvested using Qiagen Endo-Free Maxi Kits, and stored in TE Buffer or DI water at 1 to 6 µg/µL concentration, determined by a Qubit dsDNA Broad Range Kit (Invitrogen). All plasmids were acquired from Addgene unless otherwise specified:

pCAG-EGFP, pCAG-YFP, pCAG-dsRed (Addgene plasmids 11150, 11180, and 11151, respectively) [56]

pEGFP-N1 (Clontech)

pCI-tdTomato, (courtesy of Rachael Neve)

mCherry Lac-REP (Addgene plasmid 18985) [57]

Cerulean (Addgene plasmid 15214) [58]

pEAK10-His-Myc-Kal7 (Addgene plasmid 25454) [59]

pEAK10-His-Myc-Kal5 and pEAK10-His-Myc-Kal9 (Addgene plasmids 25440 and 25441, respectively) [41]

pCAG-Cerulean was constructed by removing the Cerulean gene from its native Clontech backbone [58] using the *AgeI* and *BsrGI* restriction endonucleases (New England Biolabs) and sub-cloning into the pCAG plasmid. For results in Figure 5, in all cases, concentration of fluorescent-protein-encoding plasmids was 300 ng·µL^−1^, while for the plasmids encoding Kalirin-5, Kalirin-7, and Kalirin-9, plasmid concentrations were 250, 300 and 373 ng·µL^−1^, respectively to provide equivalent molarity of delivered plasmids.

### Diffusion Measurements and Simulations

Diffusion measurements were carried out by front-loading micropipettes with samples of known concentration. Brightness was correlated to concentration using calibration curves derived from large volume (several µL) samples rear-loaded into similar micropipettes and then imaged (Fig. S1). Images were captured every minute, with fluorescence exposure occurring only during image acquisition to avoid bleaching of dyes. A tip-diffusion model was developed in MATLAB to study the recorded measurements. To generate a structural model, micropipettes were imaged under low magnification (Figure 2a) and we traced the outside of the glass to get total micropipette cross-section, which assuming longitudinal symmetry could be used to calculate total volume of the tip. Next, using the assumption of a constant ratio of outer to inner diameter of the micropipette glass we calculated the internal volume profile [60]. This internal calculated volume of the micropipette was then binned into 1 µm^3^ cubes for the purposes of simulation. To begin simulation, a sufficient number of volume bins (starting from the tip) were filled with the start concentration in order to generate a longitudinal concentration profile as shown in the middle drawing of Figure 2a. No diffusion was assumed to take place through the glass, and diffusion out of the micropipette tip was assumed to be negligible. Fick's Law was used to model diffusion:
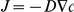
where *J* is diffusive flux, *D* is the diffusion coefficient, and 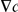 is the spatial concentration gradient of the molecular species in question. The simulation took advantage of the longitudinal axial symmetry of the micropipettes to break down simulation into two phases for each time step. First longitudinal diffusion (down the length of the micropipette) was simulated in two dimensions. Second, cross-sectional diffusion of each plane of the micropipette was carried out in two dimensions. Diffusion was calculated between each block and all adjacent blocks. Empirically determined diffusion coefficients taken from the literature were 430 µm^2^/s [61], 370 µm^2^/s [61], and 3.5 µm^2^/s [62], for Alexa Fluor 488 hydrazide, Alexa Fluor 594 hydrazide, and pEGFP-N1, respectively.

### Statistics

A one-way ANOVA test was used for cross-cell comparisons in Figure S4 and Figure 5. For individual comparisons Welch's modification on a student's t-test was used. All results are reported as mean ± s.d.

### Cost

The semi-automated system presented is built around a standard electrophysiology microscope (Nikon FN-1). Because most laboratories already have a/several micromanipulator(s) from other work (e.g. Sutter MP-285), the additional equipment needed to implement this system only costs on the order of $4000∶ $3000 for the long-travel stages and controls, $500 for the pneumatic regulators, valves and interfacing electronics, and $500 for a National Instruments DAQ card. While we used a pCIe-6259 DAQ in this work, less expensive models also work.

## Supporting Information

Figure S1 **Calibration Curve for Fluorescence Intensity vs. Concentration.** Micropipettes were rear-loaded with approximately 4 µL fluorophores at varying concentrations to generate a calibration curve for measured fluorescent intensity versus concentration. Samples were measured from a concentration of 500 µM (for the Alexa Fluors) and 500 ng·µL^−1^ (for the plasmid/SYBR Green mixture) and stepped by dilutions of two to approximately 8 µM (for the Alexa Fluors) and 8 ng·µL^−1^ (for the plasmid/SYBR Green mixture).
(TIF)

Figure S2 **Distribution of Fluorescence Emission Strength in Electroporated Cells.** (a) CA1 pyramidal cells were transfected with pCAG-EGFP using SCE, and the average fluorescence of their soma was measured at 24 hours post-transfection. Brightness was normalized to maximum possible value (2^14^ bits = 16384 values). Average normalized brightness was 0.47±0.15 (*n* = 52). (b) Cells were transfected with pCAG-YFP and their fluorescence was monitored over time in a manner similar to in part a. Values were normalized to the first data point taken at 24 hours post-transfection. Black line shows average of all normalized brightness levels (*n* = 35). At 7 days post-transfection, fluorescence intensity was 67.6% of peak value.
(TIF)

Figure S3 **Graphical User Interface (GUI) for Control.** All major controls are contained within a single window. (a) Single-cell electroporation parameters, (b) Applied SCE voltage signal (top) and measured SCE current (bottom). (c) Micropipette pressure controls, high-level controls for automated system operation, and micropipette manipulator controls. (d) Micropipette position control, micropipette clean/wash parameters, and multiwell and washing equipment position controls.
(TIF)

Figure S4 **Primary Control GUI (Detailed).** Detailed images of the portions of the control window, including (a) Single-cell electroporation parameters, (b) Applied SCE voltage signal (top) and measured SCE current (bottom). (c) Micropipette pressure controls, high-level controls for automated system operation, and manipulator controls. (d) Micropipette position control, clean/wash system parameters, and multiwell/washing equipment position control.
(TIF)

Figure S5 **Flowchart of System Operation.** Boxes are actions and processes, hexagons are preparation steps, diamonds are decision/pause points, parallelograms are data storage. “Servo1” refers to the micropipette/manipulator positioner, and “Servo2” refers to the multiwell and washing equipment positioner.
(TIF)

Figure S6 **Different Fluorophores Do Not Affect Measured Dendritic Spine Density Count.** Cells in both the CA1 (*n* = 50) and CA3 (*n* = 70) of hippocampal organotypic slices were transfected with one of the four fluorescent proteins, Cerulean, EGFP, YFP, or tdTomato, and the linear spine densities of their basal dendritic arbors were sampled (*n* = 600 dendritic spine segments). For each cell type, no significant difference exists in spine density count among the subsets of cells labeled with different fluorescent reporters (ANOVA results: *F_crit_* = 2.63, *F* = 0.29 and 0.62 for CA1 and CA3, respectively).
(TIF)

Figure S7 **Transfection of Single Cells in Acute Slices with Fluorescent Dyes.** Cells in the CA1/CA2 region of a hippocampus were transfected in short succession with Alexa Fluor 594 hydrazide (orange) and Alexa Fluor 488 hydrazide (green). Electroporation efficiency, the percentage of cells electroporated by targeting was 95.3±4.2% with mean targeting and electroporation time per cell of 26.5±8.9 seconds per cell in acute slices (*n* = 62 in five separate experiments) Scale bar 30 µm.
(TIF)

Figure S8 **Sequential Transfection of Cells with Plasmids.** CA2/CA3 cells were rapidly transfected with pCAG-EGFP using our system. At 24 hours following first-transfection 23 out of 30 cells (efficiency: 76.7%) expressed EGFP. Twelve of the expressing cells were then re-transfected by our system at 30 hours following first-transfection with a nuclear-localization-mCherry plasmid (red-arrow). 24 hours following second-transfection, cells were analyzed for expression. 100% (*n* = 11) of non re-transfected (control) cells continued to expressed EGFP. 16.7% (2/12) of re-transfected cells were no longer visible, 41.7% (4/12) were expressing both mCherry NLS and EGFP, and 50% (6/12) expressed only EGFP. Scale bar 15 µm.
(TIF)

Table S1 **Single-Cell Electroporation Efficiencies.** Measured efficiencies for different electroporation pulse parameters for a micropipette tip with approximately 8 MΩ resistance filled with 300 ng·µL^−1^ pEGFP-N1 and 50 µM Alexa Fluor 594 hydrazide in standard Ringers solution. Efficiency was determined from expression of EGFP at 24 hours post-transfection. *n* = total number of cells that were electroporated in the indicated number of independent experiments. Rise/Fall time of the micropipettes used was measured at 0.23±0.015 ms (*n* = 8).
(DOC)
