# Supplementary figures and images for: High-Throughput Single-Cell Manipulation in Brain Tissue
Source: PLoS One. 2012 Apr 20;7(4):e35603. doi: 10.1371/journal.pone.0035603 (PMC3334978; doi:10.1371/journal.pone.0035603)

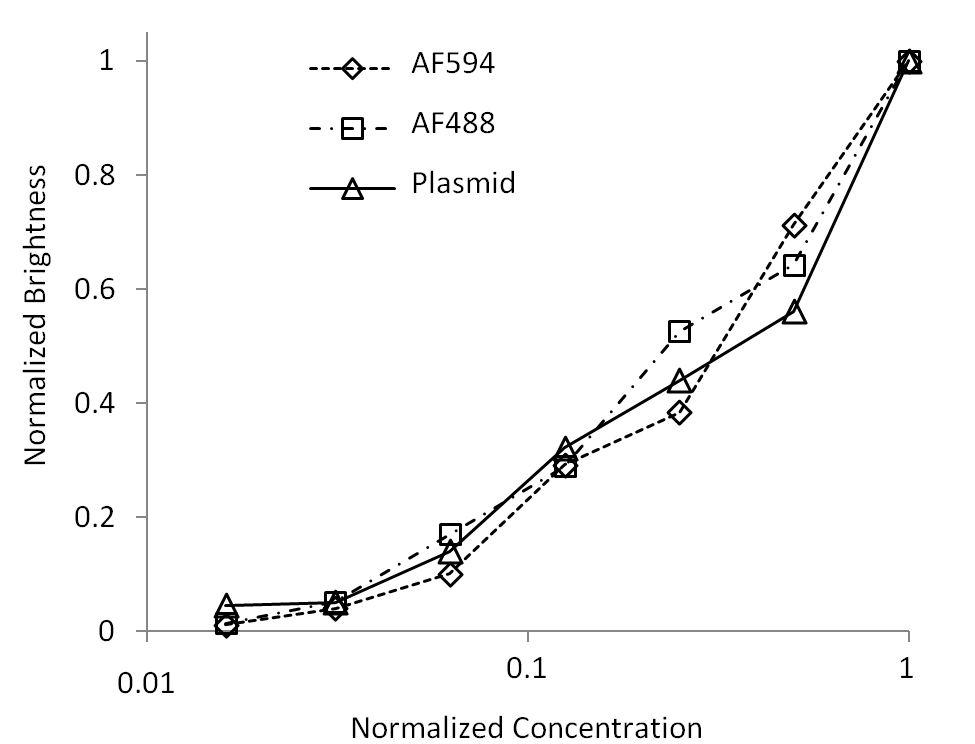

Supplement: Figure S1 — Calibration Curve for Fluorescence Intensity vs. Concentration. Micropipettes were rear-loaded with approximately 4 µL fluorophores at varying concentrations to generate a calibration curve for measured fluorescent intensity versus concentration. Samples were measured from a concentration of 500 µM (for the Alexa Fluors) and 500 ng·µL−1 (for the plasmid/SYBR Green mixture) and stepped by dilutions of two to approximately 8 µM (for the Alexa Fluors) and 8 ng·µL−1 (for the plasmid/SYBR Green mixture). (TIF) [file pone.0035603.s001.tif]

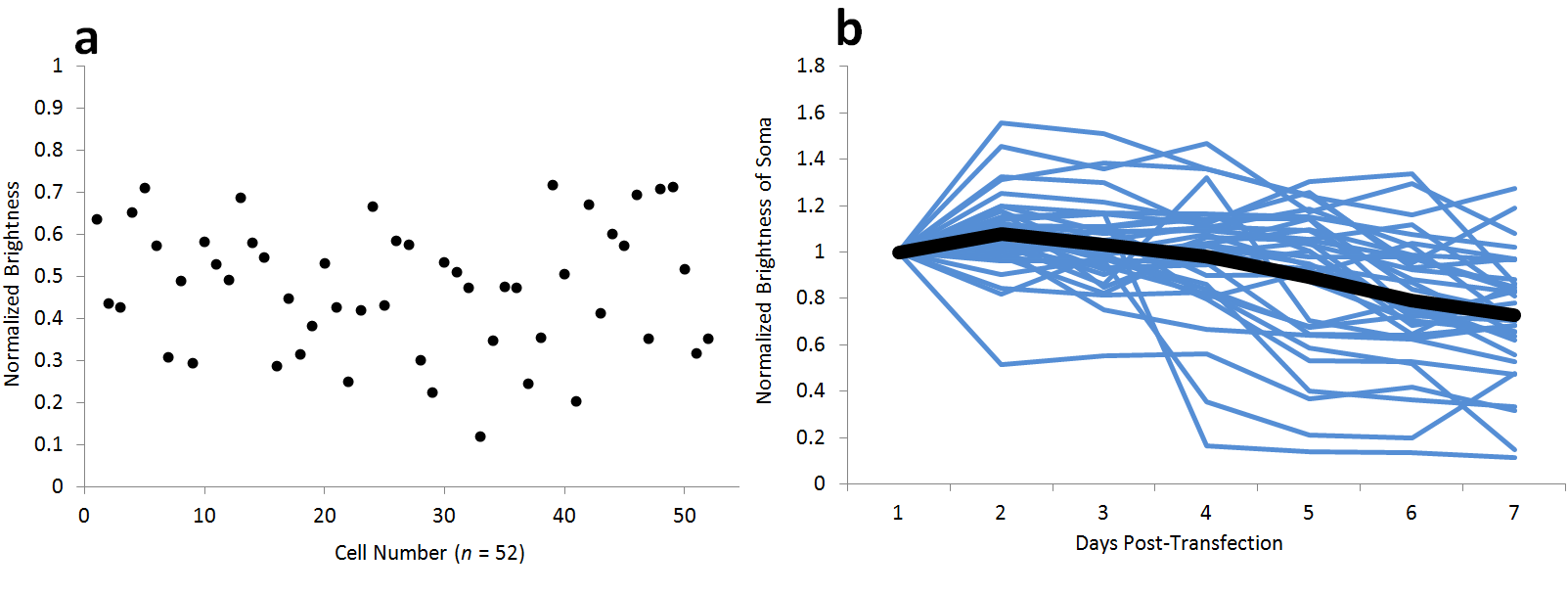

Supplement: Figure S2 — Distribution of Fluorescence Emission Strength in Electroporated Cells. (a) CA1 pyramidal cells were transfected with pCAG-EGFP using SCE, and the average fluorescence of their soma was measured at 24 hours post-transfection. Brightness was normalized to maximum possible value (214 bits = 16384 values). Average normalized brightness was 0.47±0.15 (n = 52). (b) Cells were transfected with pCAG-YFP and their fluorescence was monitored over time in a manner similar to in part a. Values were normalized to the first data point taken at 24 hours post-transfection. Black line shows average of all normalized brightness levels (n = 35). At 7 days post-transfection, fluorescence intensity was 67.6% of peak value. (TIF) [file pone.0035603.s002.tif]

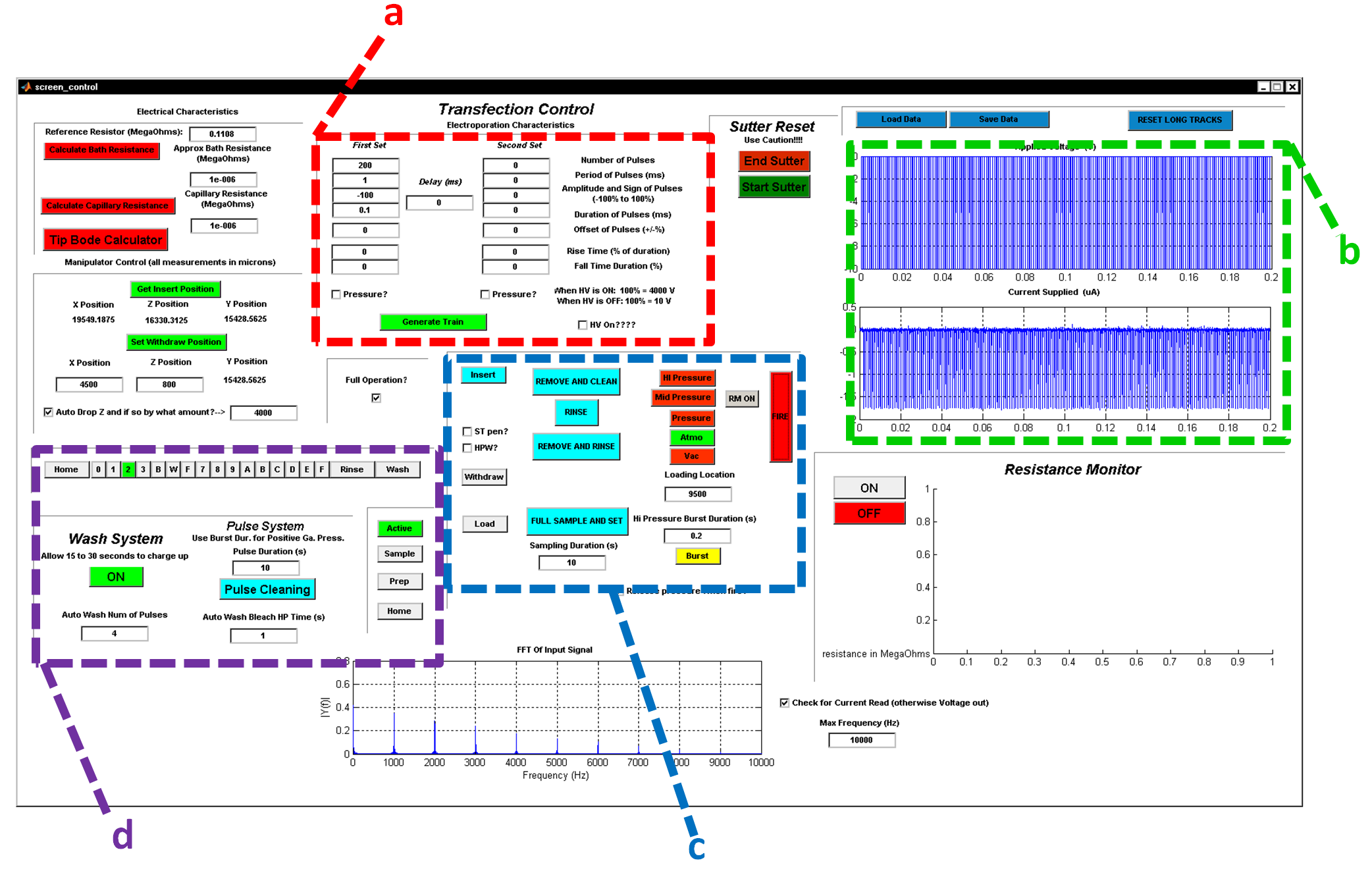

Supplement: Figure S3 — Graphical User Interface (GUI) for Control. All major controls are contained within a single window. (a) Single-cell electroporation parameters, (b) Applied SCE voltage signal (top) and measured SCE current (bottom). (c) Micropipette pressure controls, high-level controls for automated system operation, and micropipette manipulator controls. (d) Micropipette position control, micropipette clean/wash parameters, and multiwell and washing equipment position controls. (TIF) [file pone.0035603.s003.tif]

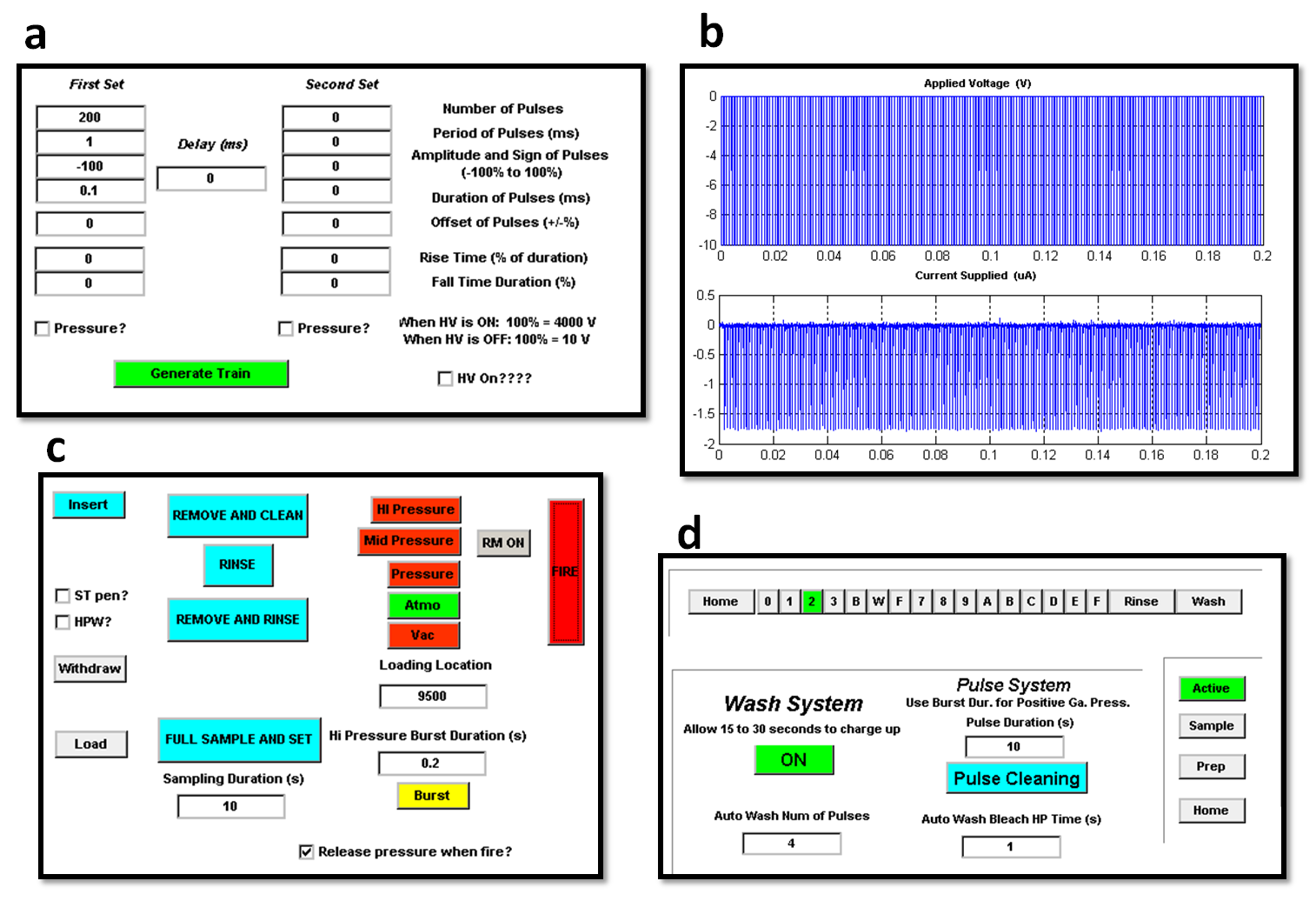

Supplement: Figure S4 — Primary Control GUI (Detailed). Detailed images of the portions of the control window, including (a) Single-cell electroporation parameters, (b) Applied SCE voltage signal (top) and measured SCE current (bottom). (c) Micropipette pressure controls, high-level controls for automated system operation, and manipulator controls. (d) Micropipette position control, clean/wash system parameters, and multiwell/washing equipment position control. (TIF) [file pone.0035603.s004.tif]

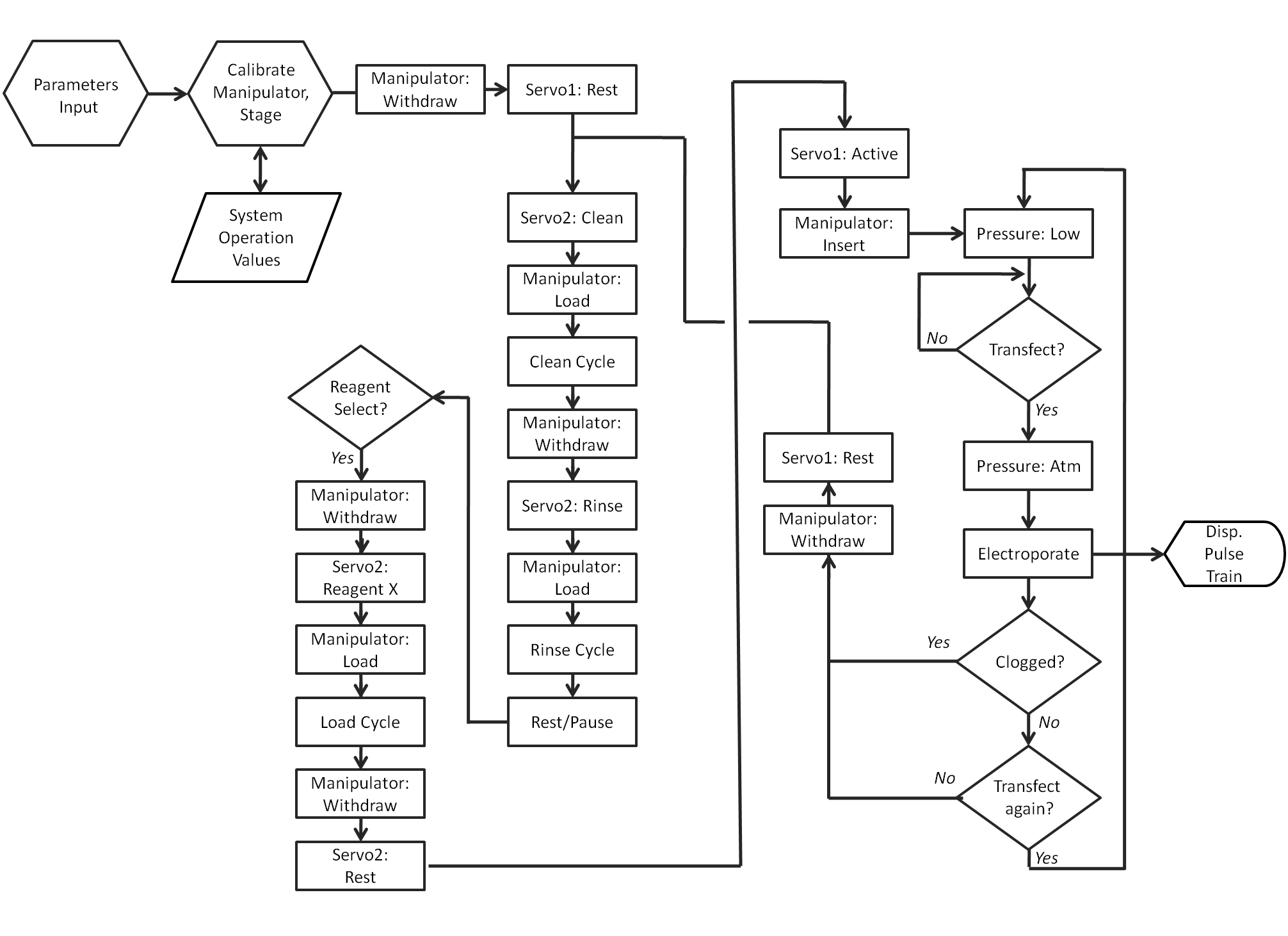

Supplement: Figure S5 — Flowchart of System Operation. Boxes are actions and processes, hexagons are preparation steps, diamonds are decision/pause points, parallelograms are data storage. “Servo1” refers to the micropipette/manipulator positioner, and “Servo2” refers to the multiwell and washing equipment positioner. (TIF) [file pone.0035603.s005.tif]

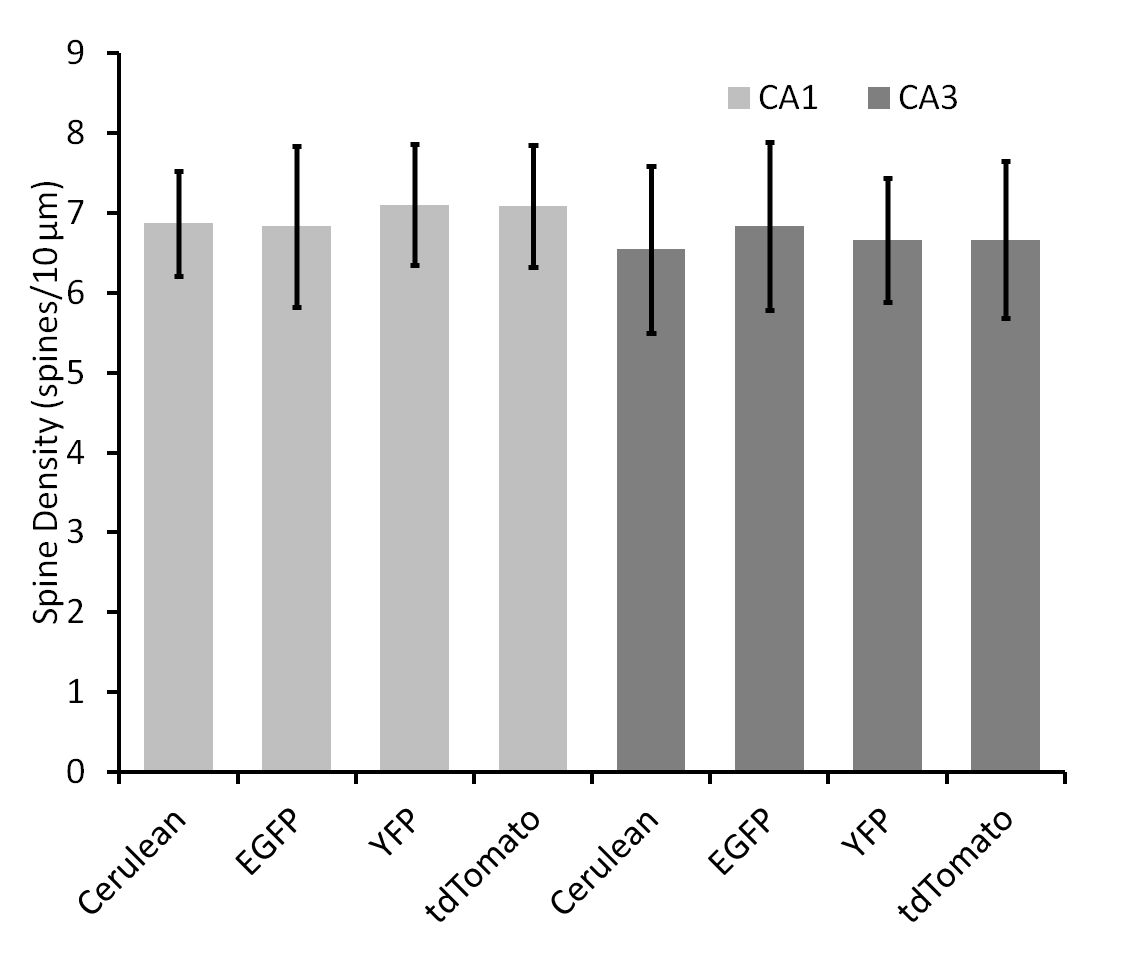

Supplement: Figure S6 — Different Fluorophores Do Not Affect Measured Dendritic Spine Density Count. Cells in both the CA1 (n = 50) and CA3 (n = 70) of hippocampal organotypic slices were transfected with one of the four fluorescent proteins, Cerulean, EGFP, YFP, or tdTomato, and the linear spine densities of their basal dendritic arbors were sampled (n = 600 dendritic spine segments). For each cell type, no significant difference exists in spine density count among the subsets of cells labeled with different fluorescent reporters (ANOVA results: Fcrit = 2.63, F = 0.29 and 0.62 for CA1 and CA3, respectively). (TIF) [file pone.0035603.s006.tif]

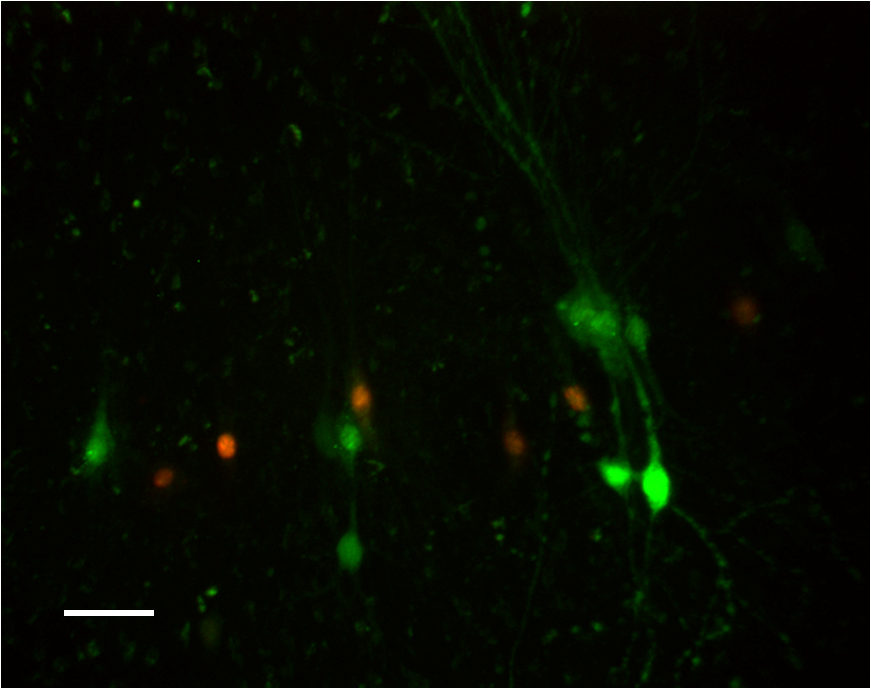

Supplement: Figure S7 — Transfection of Single Cells in Acute Slices with Fluorescent Dyes. Cells in the CA1/CA2 region of a hippocampus were transfected in short succession with Alexa Fluor 594 hydrazide (orange) and Alexa Fluor 488 hydrazide (green). Electroporation efficiency, the percentage of cells electroporated by targeting was 95.3±4.2% with mean targeting and electroporation time per cell of 26.5±8.9 seconds per cell in acute slices (n = 62 in five separate experiments) Scale bar 30 µm. (TIF) [file pone.0035603.s007.tif]

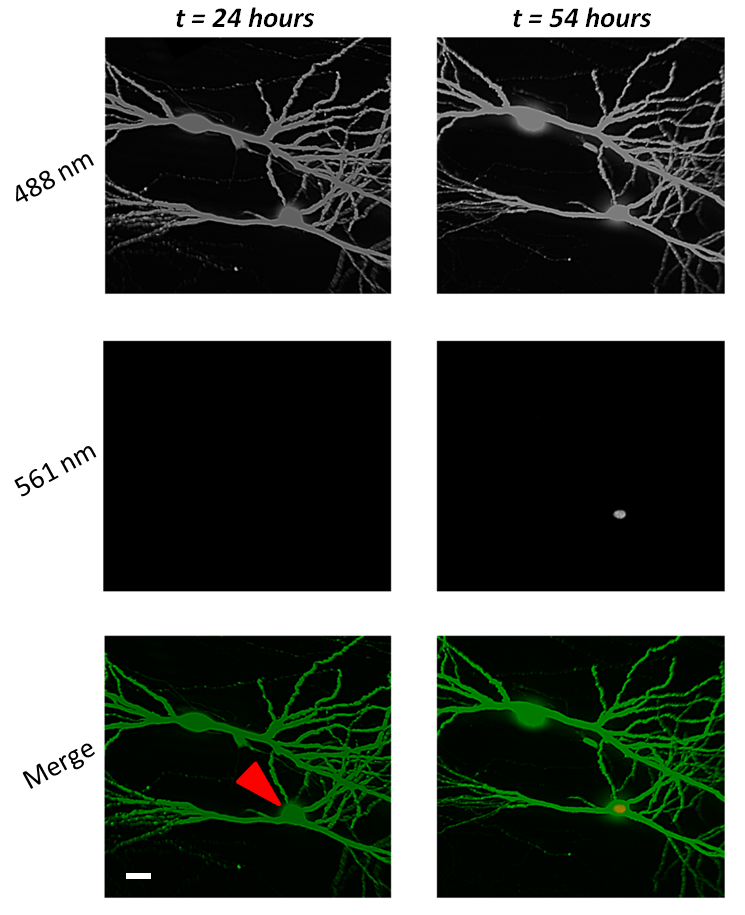

Supplement: Figure S8 — Sequential Transfection of Cells with Plasmids. CA2/CA3 cells were rapidly transfected with pCAG-EGFP using our system. At 24 hours following first-transfection 23 out of 30 cells (efficiency: 76.7%) expressed EGFP. Twelve of the expressing cells were then re-transfected by our system at 30 hours following first-transfection with a nuclear-localization-mCherry plasmid (red-arrow). 24 hours following second-transfection, cells were analyzed for expression. 100% (n = 11) of non re-transfected (control) cells continued to expressed EGFP. 16.7% (2/12) of re-transfected cells were no longer visible, 41.7% (4/12) were expressing both mCherry NLS and EGFP, and 50% (6/12) expressed only EGFP. Scale bar 15 µm. (TIF) [file pone.0035603.s008.tif]
