# Supplementary material for: High-Throughput Single-Cell Manipulation in Brain Tissue
Source: PLoS One. 2012 Apr 20;7(4):e35603. doi: 10.1371/journal.pone.0035603 (PMC3334978; doi:10.1371/journal.pone.0035603)
Supplement: Table S1 — Single-Cell Electroporation Efficiencies. Measured efficiencies for different electroporation pulse parameters for a micropipette tip with approximately 8 MΩ resistance filled with 300 ng·µL−1 pEGFP-N1 and 50 µM Alexa Fluor 594 hydrazide in standard Ringers solution. Efficiency was determined from expression of EGFP at 24 hours post-transfection. n = total number of cells that were electroporated in the indicated number of independent experiments. Rise/Fall time of the micropipettes used was measured at 0.23±0.015 ms (n = 8). (DOC) [file pone.0035603.s009.doc]

**Table S1 Single-Cell Electroporation Efficiencies.** Measured efficiencies for different electroporation pulse parameters for a micropipette tip with approximately 8 MΩ resistance filled with 300 ng·µL^-1^ pEGFP-N1 and 50µM Alexa Fluor 594 hydrazide in standard Ringers solution. Efficiency was determined from expression of EGFP at 24 hours post-transfection. *n* = total number of cells that were electroporated in the indicated number of independent experiments. Rise/Fall time of the micropipettes used was measured at 0.23±0.015 ms (*n* = 8).

| **SCE Pulse Parameters** | **Observed Efficiency** |
| --- | --- |
| **200 Hz, 20% duty cycle, -10 V, 1second** | 27.1% (8 independent experiments, n = 96) |
| **50 Hz, 2.5% duty cycle, -10 V, 1 second** | 48.7% (6 independent experiments, n = 78) |
| **50 Hz, 2.5% duty cycle, -12 V*, 1 second** | 54.8% (7 independent experiments, n = 73) |
| **50 Hz, 2.5% duty cycle, -15 V*, 1 second** | 21.6% (5 independent experiments, n = 51) |
| **50 Hz, 2.5% duty cycle, -8 V, 1 second** | 16.7% (5 independent experiments, n = 60) |
| **50 Hz, 0.5% duty cycle, -10V, 1 second** | 61.8% (6 independent experiments, n = 76) |
| **100 Hz, 1% duty cycle, -10 V, 0.5 second** | 65.6% (6 independent experiments, n = 64) |
| **100 Hz, 1% duty cycle, -10 V, 0.25 second** | 44.0% (4 independent experiments, n = 50) |
| **1 kHz, 10% duty cycle, -10 V, 0.1 second** | 81.7% (6 independent experiments, n = 93) |
| **1 kHz, 10% duty cycle, -12 V*, 0.1 second** | 58.4% (6 independent experiments, n = 89) |
| **1 kHz, 10% duty cycle, -8 V, 0.1 second** | 20.3% (5 independent experiments, n = 59) |
| **1 kHz, 10% duty cycle, -10 V, 0.05 second** | 60.1% (5 independent experiments, n = 69) |
| **1 kHz, 10% duty cycle, -10 V, 0.2 second** | 62.5% (6 independent experiments, n = 56) |

*Signals were created from an amplifier with ±18V power supply rails driven by a scaled signal from the NIDAQ card. For all -10V signals, the electrode voltage was applied directly by the NIDAQ card.
